# Comparison of Four Ni-Ti Rotary Systems: Dental Students’ Perceptions in a Multi-Center Simulated Study

**DOI:** 10.3390/dj13030097

**Published:** 2025-02-24

**Authors:** Francesco Puleio, Vincenzo Tosco, Riccardo Monterubbianesi, Rosario Pirri, Angela Alibrandi, Daria Pulvirenti, Michele Simeone

**Affiliations:** 1 Department of Biomedical and Dental Sciences and Morphofunctional Imaging, Messina University, 98121 Messina, Italy; daria2103.pulv@virgilio.it; 2 Department of Clinical Sciences and Stomatology (DISCO), Università Politecnica delle Marche, 60126 Ancona, Italy; v.tosco@pm.univpm.it (V.T.); r.monterubbianesi@univpm.it (R.M.); 3 Independent Researcher, 98124 Messina, Italy; rosariopirri97@gmail.com; 4 Department of Economics, Messina University, 98122 Messina, Italy; aalibrandi@unime.it; 5 Department of Neuroscience, Reproductive Science and Dentistry, University of Naples Federico II, 80138 Naples, Italy; michele.simeone@unina.it

**Keywords:** nickel–titanium instruments, endodontic instrument, dental education, endodontic training, student performance evaluation

## Abstract

**Background/Objectives**: Nickel–titanium (Ni-Ti) rotary instruments have significantly enhanced the efficiency and safety of root canal shaping. However, the variety of Ni-Ti systems, characterized by differences in alloy composition, taper design, and heat treatment, may influence their usability, particularly for novice operators. This study aimed to evaluate the perceptions of dental students using four Ni-Ti rotary systems (MTwo, SlimShaper Pro, ProTaper Gold, and HyFlex EDM) in a simulated environment. **Methods**: Forty dental students from two universities participated in the study and completed a standardized preoperative training session. Root canal shaping was performed on resin teeth models, and an 11-item questionnaire was used to assess various parameters, including flexibility, ease of use, and the ability to maintain working length. **Results**: The results indicated significant differences in student feedback. SlimShaper Pro and HyFlex EDM were preferred for their flexibility, reduced resistance, and ease of instrument transitions, likely due to their smaller tapers and martensitic alloys. MTwo was rated the least flexible and more challenging to use. **Conclusions:** These findings underline the importance of user-friendly Ni-Ti systems in pre-clinical training to reduce procedural difficulties and operator stress. Further research should validate these findings in clinical settings to improve the training of novice operators.

## 1. Introduction

Patient care simulation is crucial for undergraduate students, including practicing theoretical knowledge and retaining it in an applicable clinical context to achieve competence in everyday practice. This teaching method presents a solid body of literature, making patient care simulation an effective way to teach medical protocols to undergraduate students. Simulation training is also an effective way to help students’ stress-managing skills. Lack of experience, dexterity, technical proficiency, and patient communication can make the inexperienced clinician develop anxiety and stress regarding operative procedures, thus increasing uneasiness [1,2,3].

Hands-on activities and courses can help students understand clinical reflections of basic biomedical sciences, keeping them engaged, intellectually stimulated, and motivated, thus improving their critical thinking skills and operative autonomy [4]. These positive outcomes in students’ learning experience have been researched and tested in almost all fields of dentistry, including implantology, oral pathology, and operative dentistry [5,6,7]. Case-based teaching methods can also improve students’ learning experience in the understanding of dental materials science [8,9,10]. Model- and case-based teaching is useful not only for clinical skills but also extra-clinical abilities.

In endodontics, accuracy and, consequently, its practical teaching are crucial, as evaluated based on analyses of undergraduate students’ endodontic performance on patients that evidence the need for better practical teaching using models of simulated endodontic systems [11,12,13,14,15,16].

Inexperienced operators facing endodontic treatments may encounter difficulties because of the significant differences among the various rotary instrumentation systems available on the market [17]. Ni-Ti endodontic instruments have undergone—and continue to undergo—constant evolution, both in terms of materials and the resulting number of instruments used, as well as the proper technique for guiding them to the correct working length [18]. Similarly, irrigation and activation techniques have also undergone evolution and improvement [19].

The new generations of endodontic instruments, for example, rely on thermal treatment of the alloy to enhance its mechanical characteristics [20]. Compared to conventional Ni-Ti and M-wire instruments, thermally treated instruments have demonstrated better flexibility and fatigue resistance, particularly those subjected to blue and gold heat treatments [21]. In beginners’ hands, the difference in operative outcomes can be attributed to the nature of the NiTi alloy: austenitic NiTi instruments, being more rigid, are more effective in shaping, whereas martensitic NiTi instruments, due to their greater flexibility, cause fewer alterations to the canal’s anatomy [22,23].

Moreover, numerous endodontic instrument sequences are commercially available, each offering various features beyond thermal treatment, including, for instance, different tapers. Conservative endodontic approaches advocate for smaller access cavities and minimal root canal shaping, with apical diameters ranging from 0.2 mm to 0.4 mm and tapers strictly below 6%, as a strategy to reduce the likelihood of root fractures, although it may not guarantee optimal cleaning and shaping [24,25,26]. On the other hand, using instruments with larger tapers can enhance the removal of infected tissue and improve irrigant penetration, but it may compromise the tooth’s structure and increase the risk of complications, such as perforations, ledges, canal transportation, and microcrack formation [27,28].

The objective of this research is to evaluate students’ responses to four different shaping sequences built in four Ni-Ti alloys (M-wire, Gold, CM, and Dual Wire) and with different characteristics in a simulated root canal. This study focused on four widely used systems—MTwo (VDW, Munich, Germany), SlimShaper Pro (Zarc4endo, Gijón, Asturias, Spain), ProTaper Gold (Dentsply-Sirona, Baillagues, Switzerland), and HyFlex EDM (Coltene-Whaledent, Altstätten, Switzerland)—chosen to represent a diverse range of metallurgical compositions and design features.

MTwo, composed of an austenitic alloy, was selected for its rigidity and traditional design, which contrasts with the martensitic alloys used in the other systems. SlimShaper Pro and HyFlex EDM were chosen due to their advanced thermal treatments, which enhance flexibility and reduce fatigue. ProTaper Gold, with its proprietary gold heat treatment, was included to evaluate its balance between flexibility and cutting efficiency. These systems were selected to cover a spectrum of mechanical and operational characteristics, providing a comprehensive evaluation of their usability and performance for dental students in a simulated training environment. Modern endodontic training employs a variety of simulation techniques to enhance student learning. These include virtual reality simulators, which offer immersive and interactive experiences, and haptic devices that provide tactile feedback to mimic the feel of real clinical procedures. Among the most commonly used tools are plastic teeth, which serve as an accessible and standardized medium for practicing root canal procedures. Plastic teeth replicate anatomical structures and allow students to familiarize themselves with the nuances of shaping and instrumentation without the variability and risks associated with real clinical cases. These simulation methods play a crucial role in developing technical skills and confidence among novice operators, ensuring they are better prepared for clinical challenges.

The null hypothesis is that students do not have a preferred sequence for shaping, demonstrating that the difference in the instruments does not impact shaping experience and outcomes.

## 2. Materials and Methods

### 2.1. Study Design

An anonymous questionnaire was designed by an experienced endodontist to evaluate students’ perceptions and performance with the four tested Ni-Ti instrument systems. The questionnaire included a total of 11 questions, structured to assess key parameters related to the shaping experience. These questions encompassed 2 binary items and 9 Likert-scale ratings (1 = very low, 5 = very high), covering aspects like instrument flexibility, ease of use, screw-in effect, and overall clinical usability. The full questionnaire is provided below in Table 1.

The term “overall simplicity” was defined as the perceived ease of performing the entire shaping process using each instrument system. This parameter accounted for factors like operational stability, tactile feedback, and the physical effort required to complete the procedure. The distinction between “simplicity of transitioning” and “difficulties in maintaining working length” was clarified as follows: “simplicity of transitioning” evaluated the ergonomic and sequential logic of using consecutive instruments, while “difficulties in maintaining working length” focused on the technical challenges of keeping the file at the desired depth throughout the shaping process.

### 2.2. Recruitment of Participants

A total of 40 students were randomly recruited for this study: 20 students from the University of Messina and 20 students from the Università Politecnica delle Marche. All participants were in their 5th or 6th year of undergraduate dental studies and had completed theoretical and pre-clinical training in endodontics. Recruitment was conducted independently at each university to ensure a diverse sample of participants. Informed consent was obtained from all participants prior to their involvement in the study. Participants were made aware that their inclusion in the study was entirely voluntary, and they were assured that the data collected would remain anonymous and be used solely for research purposes. The anonymity of the questionnaire was emphasized to protect their privacy and encourage honest responses.

### 2.3. Preoperative Training

All participants attended a 45 min preoperative training session designed to ensure a thorough understanding of the instruments and procedures involved. This session aimed to create a uniform baseline of knowledge and skills among participants, thus minimizing variability due to differences in prior exposure to Ni-Ti rotary systems. During the training, students were introduced to the unique features of each of the four Ni-Ti systems: MTwo, SlimShaper Pro, ProTaper Gold, and HyFlex EDM. The instructors explained the design, metallurgical properties, and shaping sequences of each system, highlighting the thermal treatments applied to enhance flexibility and fatigue resistance. Visual aids, including detailed diagrams and instructional videos, were used to illustrate these concepts and enhance understanding. Operational parameters, such as the recommended torque and rotational speeds for each instrument, were discussed in depth. Students were instructed on the appropriate settings for each system, ensuring consistent application during the experiment to prevent procedural errors and instrument failures. A hands-on practice session followed the theoretical training, where students performed shaping on resin blocks under close supervision. This practical component allowed participants to familiarize themselves with the tactile feedback and operational nuances of each instrument in a controlled environment. The instructors provided real-time feedback, addressing any questions and reinforcing proper techniques. Finally, the students were briefed on the evaluation questionnaire they would complete after each procedure. They were encouraged to provide honest and thoughtful responses based on their experiences, reassured that the questionnaire was entirely anonymous. This training session ensured that all participants were adequately prepared and confident in performing the procedures, allowing the study to focus on the comparative evaluation of the instruments themselves. To ensure a standardized preoperative training experience across both universities, the same PowerPoint presentation was used to deliver theoretical instruction on the characteristics and use of the Ni-Ti rotary systems. Although the training sessions were conducted by different instructors at each university, they followed the same structured presentation and adhered to uniform teaching guidelines. This approach minimized potential variability in knowledge acquisition and guaranteed that all students received identical theoretical and practical instructions before performing the shaping procedures. During the pre-clinical phase, an expert operator conducted the training session, supported by two additional tutors who assisted the students during the shaping procedures. The same approach was followed at both universities, ensuring consistency in supervision and guidance. However, the sessions were held at different times at each institution, rather than with all students present simultaneously.

### 2.4. Shaping Sessions and Experience Evaluation

**Figure 1:**
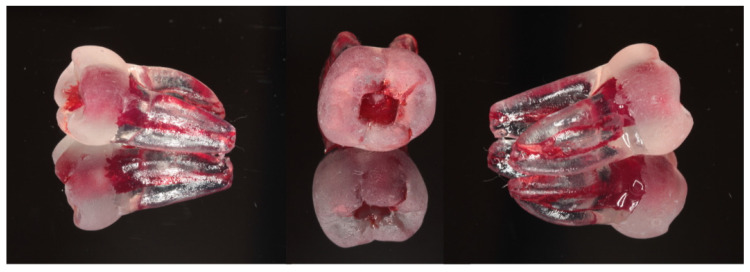
Resin tooth model used.

After completing the preoperative training, students proceeded with the root canal shaping procedures using simulated resin teeth. The resin models were standardized replicas of mandibular first molars (Surpreendente 3D Tooth, Vila Nova de Gaia, Porto, Portugal) designed to ensure uniformity across all samples, visible in Figure 1.

The tooth model featured two canals in the mesial root and one canal in the distal root. An X-Smart Plus motor (Dentsply Sirona, Ballaigues, Switzerland) was used, with the RPM and torque values manually adjusted for each instrument used. Each participant received four resin teeth, one for each instrument system tested: MTwo, SlimShaper Pro, ProTaper Gold, and HyFlex EDM. The order in which the instruments were tested was randomized for each student to minimize potential sequence-related bias. Following the manufacturer’s recommendations, students adhered to the specified shaping sequences and operational parameters for torque and rotational speed. Supervisors were present to ensure procedural adherence and address technical issues only if absolutely necessary, allowing participants to independently assess the instruments’ performance. The shaping process was conducted until the final instrument in the sequence, with an apical diameter of ISO #25, was reached:

MTwo: 10/0.04, 15/0.05, 20/0.06, 25/0.06;
SlimShaper Pro: 15/0.06-2, 20/0.03-4, 25/0.03-4;
ProTaper Gold: 18/0.02v, 20/0.04v, 20/0.07v, 25/0.08v;
HyFlex EDM: 15/0.03, 10/0.05, 20/0.05, 25/0.08v.

Upon completing the shaping procedures with each instrument, students were asked to complete the evaluation questionnaire.

### 2.5. Data Collection and Statistical Analysis

Data were processed and analyzed by an independent researcher blinded to the instrument assignments. This ensured that the evaluation of results was free from potential bias and that the findings accurately reflected the participants’ feedback. Descriptive statistics, including the mean and the standard deviation, were calculated for each parameter across the four instrument systems. To assess the normality of the sample’s distribution, the Shapiro–Wilk test was performed prior to conducting the ANOVA test. This test is commonly used for small to moderate sample sizes, and it evaluates whether the data follow a normal distribution. The results confirmed that the data met the assumptions for parametric testing, allowing us to proceed with ANOVA. A one-way analysis of variance (ANOVA) was performed to determine whether statistically significant differences existed among the instruments, with statistical significance set at *p* < 0.05. After applying ANOVA, Tukey’s post hoc test was conducted for comparisons that showed statistical significance to determine which specific sequences differed significantly from each other. To account for the increased likelihood of errors in multiple comparisons, the Bonferroni correction was applied. This method adjusts the alpha level by dividing it by the number of possible pairwise comparisons. For four groups, the number of pairwise comparisons is six, resulting in an adjusted alpha level of 0.008. Consequently, in this analysis, only *p*-values less than 0.008 were considered statistically significant. All statistical analyses were conducted using SPSS software (version 30.0.0, IBM, Armonk, NY, USA).

## 3. Results

### 3.1. Descriptive Statistics

The mean and the standard deviation for each question and instrument were analyzed to provide a comprehensive overview of the participants’ evaluations. Below are the results for all 11 questions based on the participants’ responses.

(1) Question 1: Were you able to complete the entire shaping sequence?
  All students successfully completed the shaping sequence with all four instrument systems. No variability was observed, and, therefore, no mean or standard deviation was calculated.
(2) Question 7: Did any of the instruments fracture inside the canal?
  All participants reported no fractures for any instrument. No variability was observed, and, therefore, no mean or standard deviation was calculated.

The mean and standard deviation values are grouped in Table 2.

### 3.2. Inferential Statistics

A one-way ANOVA test was performed to determine whether significant differences existed among the four Ni-Ti rotary systems for the analyzed questions. Statistically significant differences were identified for the following questions (*p* < 0.05): Questions 2, 3, 5, 8, and 11. For the remaining questions, no statistically significant differences were observed.

For the questions where ANOVA indicated significant differences, Tukey’s post hoc test was performed to identify pairwise differences among the instruments.

- Question 2: How would you rate the flexibility of the instruments during shaping?
  SlimShaper Pro, ProTaper Gold, and HyFlex EDM were rated as significantly more flexible than MTwo (*p* < 0.008).
- Question 3: How would you rate the overall simplicity of using these instruments during the shaping process?
  SlimShaper Pro, ProTaper Gold, and HyFlex EDM were rated as significantly easier to use compared to MTwo (*p* < 0.008).
- Question 5: How would you rate the simplicity of transitioning from one instrument to the next during the shaping process while maintaining the working length?
  SlimShaper Pro was rated as significantly simpler to use than MTwo, ProTaper Gold, and HyFlex EDM (*p* < 0.008).
- Question 8: Did you encounter difficulties in reaching the working length?
  Students reported significantly more difficulty with ProTaper Gold compared to HyFlex EDM (*p* < 0.008).
- Question 11: Would you use this sequence of instruments in a real clinical case?
  SlimShaper Pro and HyFlex EDM were rated as significantly more suitable for clinical use than MTwo (*p* < 0.008).

## 4. Discussion

Since their introduction, nickel–titanium (Ni-Ti) instruments have revolutionized endodontic practice, making root canal shaping procedures more efficient, predictable, and safe [29]. Over the years, the market has seen the development of a wide variety of Ni-Ti systems, each with unique characteristics, including differences in diameters, alloy compositions, and blade designs [30,31]. The question of which Ni-Ti instruments are the easiest to use for students and less experienced practitioners remains unclear, highlighting the need for studies like this one to guide instrument selection for educational and clinical purposes [32]. To address this gap in knowledge, we conducted a comparative study evaluating the perceptions and performance of dental students using four different Ni-Ti rotary systems in a simulated environment. This type of research, which leverages a pre-clinical simulation, is widely validated in the literature as a reliable method for assessing the usability and effectiveness of dental instruments. Simulated models provide a controlled and standardized setting that eliminates variability associated with clinical cases, allowing for a focused comparison of instrument performance [30,31]. Moreover, simulation-based studies are particularly useful in educational contexts, as they help identify tools that can enhance the learning experience for students while minimizing procedural risks during their initial clinical exposure [33]. The use of standardized resin teeth mimicking the anatomy of a mandibular first molar further ensures reproducibility and consistency, as highlighted in previous studies validating the use of such models for pre-clinical training in endodontics [31,33].

The instruments used in this study represent a diverse range of nickel–titanium (Ni-Ti) systems, each characterized by unique metallurgical properties, taper designs, and mechanical behaviors. These differences are critical in shaping performance and operator experience [34,35]. These differences in alloy composition, taper design, and mechanical behavior are reflected in the questionnaire responses provided by the students. The varying characteristics of the instruments resulted in distinct preferences and perceptions, highlighting how each system’s specific features influenced the shaping experience. The diverse feedback underscores the importance of understanding how instrument design impacts usability, especially for less experienced operators, and validates the need for comparative studies like this one to guide the selection of tools in both educational and clinical contexts. All recruited students were able to complete the shaping sequence with all four tested instruments. The unanimous completion of the shaping sequence by all participants suggests that the students were well-prepared and adequately trained during the preoperative session. The standardized training likely played a critical role in familiarizing the students with the operational parameters and handling techniques of each instrument system, thereby minimizing procedural difficulties. Finally, the use of standardized resin teeth, which offer uniform canal anatomy, may have contributed to the absence of significant challenges during the shaping procedures. This controlled environment eliminates anatomical variability, ensuring that the focus remains on the instruments’ performance and the students’ technique.

The results revealed significant differences in how students perceived the instruments. Therefore, this finding directly refutes the null hypothesis, highlighting the influence of instrument choice on user performance. MTwo was rated as the least flexible and the most difficult to use compared to SlimShaper Pro, ProTaper Gold, and HyFlex EDM. These perceptions align with the metallurgical properties of the instruments; MTwo is manufactured using an austenitic alloy, which is inherently less flexible than the martensitic alloys used in the other three systems [36]. Increased flexibility, a hallmark of martensitic instruments, not only allows for better adaptation to curved canals but also enhances tactile feedback, which is particularly beneficial for inexperienced operators. This likely explains why students found SlimShaper Pro, ProTaper Gold, and HyFlex EDM easier to use.

The effectiveness of the SlimShaper Pro in maintaining the working length and easing transitions between instruments can be explained by its smaller final taper (ranging between 3% and 4%) [37]. Instruments with smaller tapers exert less lateral pressure against the canal walls, thereby reducing torsional stress and the likelihood of binding [38]. In contrast, instruments with larger tapers, such as MTwo (25/0.06), ProTaper Gold (25/0.08), and HyFlex EDM (25/0.08v), engage a larger surface area of the canal walls, which increases frictional resistance. For novice operators, this increased resistance may complicate the transition between instruments, leading to a higher cognitive and manual workload. Consequently, smaller taper systems are more forgiving during shaping procedures, especially in curved or narrow canals, which supports their suitability for less experienced clinicians [39]. A minimal approach to root canal preparation has been proposed to lower the likelihood of root fracture; however, this can result in inadequate cleaning and shaping. Conversely, using instrumentation with a larger taper may aid in removing infected tissue, but it could also compromise the tooth’s structural integrity [40]. In terms of reaching the working length, HyFlex EDM performed better than ProTaper Gold, likely due to differences in the design of the initial instruments in each sequence. HyFlex EDM starts with a 15/0.03 taper, providing less aggressive shaping, whereas ProTaper Gold begins with a 17/0.03–0.09 taper, which may create greater resistance in tighter or curved canals. This observation highlights how instrument geometry can influence shaping performance, particularly for students who are still developing their clinical dexterity. Finally, when asked whether they would use these instrument sequences in real clinical cases, students showed a clear preference for SlimShaper Pro and HyFlex EDM over MTwo. This preference likely reflects the perceived advantages of flexibility, ease of use, and smoother transitions during shaping. These findings emphasize the importance of tailoring instrument selection to the operator’s skill level and ensuring that educational protocols prioritize systems that offer a balance of performance and usability. The findings of this study suggest that inexperienced operators tend to prefer instruments with smaller diameters and martensitic alloys. For beginner practitioner, features like high flexibility and less aggressive tapers are crucial, as they provide a more forgiving learning experience and help build confidence in handling rotary systems [41]. Consequently, incorporating instruments with these characteristics in pre-clinical training programs may enhance the learning curve, minimize operator stress, and foster greater procedural success during early clinical practice. This study highlights the critical factors influencing the usability of Ni-Ti rotary systems in an educational context. Flexibility, taper design, and sequence simplicity emerged as key determinants of student performance and preferences. By identifying these differences, the findings provide valuable guidance for optimizing instrument selection in both educational and clinical settings, ultimately contributing to improved operator proficiency and patient outcomes.

### Limitations

This study has several limitations that should be acknowledged. First, the sample size was relatively small and limited to students from two universities, which may affect the generalizability of the findings. Second, the use of resin teeth, while standardized and effective for controlled comparisons, does not fully replicate the complexity of natural root canal anatomy, including variations in canal curvature and dentin hardness. Additionally, the evaluation was based on self-reported perceptions, which, although valuable, may introduce subjective bias.

In this study, we focused exclusively on the students’ subjective perceptions of each Ni-Ti system, such as whether they managed to complete the shaping sequence, if the instrument became stuck or fractured, and the ease of maintaining working length. We did not perform any objective assessments (e.g., micro-CT or morphological analyses) to evaluate the actual canal preparation outcome. Our primary aim was to explore and compare students’ experiences in a pre-clinical educational context rather than measuring clinical or radiographic parameters.

Another significant limitation of this study is the potential bias introduced by the prior knowledge of the students about endodontics and the specific rotary systems tested. While all participants underwent standardized preoperative training, their familiarity with the theoretical and practical aspects of the instruments may have influenced their perceptions and performance. A blinded approach, where students are unaware of the specific systems being used, could reduce this bias but was not feasible within the scope of this study. Future research should consider employing blinded study designs to minimize operator-related biases and improve the objectivity of the findings.

Lastly, this study did not assess long-term performance or clinical outcomes, which are essential for understanding the full impact of Ni-Ti rotary instruments in real-world practice. Future research should address these limitations by incorporating larger and more different samples, using natural teeth, and evaluating the instruments in clinical settings.

## 5. Conclusions

Considering the study’s findings and limitations, it is clear that the choice of Ni-Ti rotary systems can influence novice operators’ learning experience. The students in this investigation reported that instruments with smaller tapers and martensitic alloys offered greater perceived flexibility, less resistance during use, and smoother transitions between files. Specifically, SlimShaper Pro and HyFlex EDM were judged by participants to be more user-friendly and clinically applicable than MTwo, which was perceived to be less flexible and more difficult to manage.

It is important to highlight that these observations are based on subjective, self-reported feedback collected in a simulated environment and do not constitute an objective evaluation of clinical efficacy. Future research should integrate more objective assessments (e.g., canal morphology analysis, long-term clinical outcomes, and blinded testing) and consider the use of natural teeth to better understand the true clinical impact of these Ni-Ti systems.

## Figures and Tables

**Table 1:** Questionnaire submitted to students.

Question	MTwo	SlimShaper Pro	ProTaper Gold	HyFlex EDM
Question 1: Were you able to complete the entire shaping sequence?	□ Yes □ No	□ Yes □ No	□ Yes □ No	□ Yes □ No
Question 2: How would you rate the flexibility of the instruments during shaping?	□ 1 □ 2 □ 3 □ 4 □ 5	□ 1 □ 2 □ 3 □ 4 □ 5	□ 1 □ 2 □ 3 □ 4 □ 5	□ 1 □ 2 □ 3 □ 4 □ 5
Question 3: How would you rate the overall simplicity of using these instruments during the shaping process?	□ 1 □ 2 □ 3 □ 4 □ 5	□ 1 □ 2 □ 3 □ 4 □ 5	□ 1 □ 2 □ 3 □ 4 □ 5	□ 1 □ 2 □ 3 □ 4 □ 5
Question 4: How would you rate the screw-in effect of the instruments?	□ 1 □ 2 □ 3 □ 4 □ 5	□ 1 □ 2 □ 3 □ 4 □ 5	□ 1 □ 2 □ 3 □ 4 □ 5	□ 1 □ 2 □ 3 □ 4 □ 5
Question 5: How would you rate the simplicity of transitioning from one instrument to the next during the shaping process while maintaining the working length?	□ 1 □ 2 □ 3 □ 4 □ 5	□ 1 □ 2 □ 3 □ 4 □ 5	□ 1 □ 2 □ 3 □ 4 □ 5	□ 1 □ 2 □ 3 □ 4 □ 5
Question 6: Did any of the instruments get stuck inside the canal?	□ 1 □ 2 □ 3 □ 4 □ 5	□ 1 □ 2 □ 3 □ 4 □ 5	□ 1 □ 2 □ 3 □ 4 □ 5	□ 1 □ 2 □ 3 □ 4 □ 5
Question 7: Did any of the instruments fracture inside the canal?	□ Yes □ No	□ Yes □ No	□ Yes □ No	□ Yes □ No
Question 8: Did you encounter difficulties in reaching the working length?	□ 1 □ 2 □ 3 □ 4 □ 5	□ 1 □ 2 □ 3 □ 4 □ 5	□ 1 □ 2 □ 3 □ 4 □ 5	□ 1 □ 2 □ 3 □ 4 □ 5
Question 9: Did you encounter difficulties in maintaining the working length?	□ 1 □ 2 □ 3 □ 4 □ 5	□ 1 □ 2 □ 3 □ 4 □ 5	□ 1 □ 2 □ 3 □ 4 □ 5	□ 1 □ 2 □ 3 □ 4 □ 5
Question 10: Did you notice an excessive rotational speed that caused you difficulties during the shaping process?	□ 1 □ 2 □ 3 □ 4 □ 5	□ 1 □ 2 □ 3 □ 4 □ 5	□ 1 □ 2 □ 3 □ 4 □ 5	□ 1 □ 2 □ 3 □ 4 □ 5
Question 11: Would you use this sequence of instruments in a real clinical case?	□ 1 □ 2 □ 3 □ 4 □ 5	□ 1 □ 2 □ 3 □ 4 □ 5	□ 1 □ 2 □ 3 □ 4 □ 5	□ 1 □ 2 □ 3 □ 4 □ 5

**Table 2:** Mean and standard deviation of the answers to the questionnaire. Statistically significant results (*p* < 0.008) are highlighted. Values statistically higher are marked in green. Values statistically lower are marked in red.

Question	MTwo (Mean ± SD)	SlimShaper Pro (Mean ± SD)	ProTaper Gold (Mean ± SD)	HyFlex EDM (Mean ± SD)
Question 2: How would you rate the flexibility of the instruments during shaping?	2.20 ± 1.28 (*p* < 0.008)	3.70 ± 0.65 (*p* < 0.008)	3.30 ± 1.03 (*p* < 0.008)	3.80 ± 1.00 (*p* < 0.008)
Question 3: How would you rate the overall simplicity of using these instruments during the shaping process?	2.95 ± 0.60 (*p* < 0.008)	3.80 ± 0.61 (*p* < 0.008)	3.70 ± 0.47 (*p* < 0.008)	3.90 ± 0.71 (*p* < 0.008)
Question 4: How would you rate the screw-in effect of the instruments?	3.20 ± 1.50	2.60 ± 0.88	2.70 ± 1.12	2.20 ± 0.89
Question 5: How would you rate the simplicity of transitioning from one instrument to the next during the shaping process while maintaining the working length?	2.60 ± 1.15 (*p* < 0.008)	4.45 ± 0.50 (*p* < 0.008)	2.80 ± 1.03 (*p* < 0.008)	3.25 ± 0.88 (*p* < 0.008)
Question 6: Did any of the instruments get stuck inside the canal?	1.95 ± 1.14	1.40 ± 0.50	1.70 ± 1.03	1.60 ± 0.88
Question 8: Did you encounter difficulties in reaching the working length?	2.10 ± 1.17	2.20 ± 1.28	2.50 ± 1.31 (*p* < 0.008)	1.40 ± 0.50 (*p* < 0.008)
Question 9: Did you encounter difficulties in maintaining the working length?	2.10 ± 1.17	1.90 ± 0.96	1.95 ± 0.88	2.30 ± 1.30
Question 10: Did you notice an excessive rotational speed that caused you difficulties during the shaping process?	2.70 ± 1.38	2.30 ± 0.80	2.30 ± 1.38	2.30 ± 1.30
Question 11: Would you use this sequence of instruments in a real clinical case?	2.75 ± 1.59 (*p* < 0.008)	4.05 ± 0.88 (*p* < 0.008)	3.30 ± 0.91	3.53 ± 1.22 (*p* < 0.008)

## Data Availability

The original contributions presented in this study are included in the article. Further inquiries can be directed to the corresponding author.
